# FKL-Spa-LapRLS: an accurate method for identifying human microRNA-disease association

**DOI:** 10.1186/s12864-018-5273-x

**Published:** 2018-12-31

**Authors:** Limin Jiang, Yongkang Xiao, Yijie Ding, Jijun Tang, Fei Guo

**Affiliations:** 1 School of Computer Science and Technology, College of Intelligence and Computing, Tianjin University, Tianjin, China; 2 Tianjin University Institute of Computational Biology, Tianjin University, Tianjin, China; 3 School of Chemical Engineering and Technology, Tianjin University, Tianjin, China; 4 School of Electronic and Information Engineering, Suzhou University of Science and Technology, Suzhou, China; 5 Department of Computer Science and Engineering, University of South Carolina, Columbia, SC USA

**Keywords:** MiRNA-disease association, Similarity kernel, Fast kernel learning, Sparse kernel, Laplacian regularized least squares

## Abstract

**Background:**

In the process of post-transcription, microRNAs (miRNAs) are closely related to various complex human diseases. Traditional verification methods for miRNA-disease associations take a lot of time and expense, so it is especially important to design computational methods for detecting potential associations. Considering the restrictions of previous computational methods for predicting potential miRNAs-disease associations, we develop the model of FKL-Spa-LapRLS (Fast Kernel Learning Sparse kernel Laplacian Regularized Least Squares) to break through the limitations.

**Result:**

First, we extract three miRNA similarity kernels and three disease similarity kernels. Then, we combine these kernels into a single kernel through the Fast Kernel Learning (FKL) model, and use sparse kernel (Spa) to eliminate noise in the integrated similarity kernel. Finally, we find the associations via Laplacian Regularized Least Squares (LapRLS). Based on three evaluation methods, global and local leave-one-out cross validation (LOOCV), and 5-fold cross validation, the AUCs of our method achieve 0.9563, 0.8398 and 0.9535, thus it can be seen that our method is reliable. Then, we use case studies of eight neoplasms to further analyze the performance of our method. We find that most of the predicted miRNA-disease associations are confirmed by previous traditional experiments, and some important miRNAs should be paid more attention, which uncover more associations of various neoplasms than other miRNAs.

**Conclusions:**

Our proposed model can reveal miRNA-disease associations and improve the accuracy of correlation prediction for various diseases. Our method can be also easily extended with more similarity kernels.

**Electronic supplementary material:**

The online version of this article (10.1186/s12864-018-5273-x) contains supplementary material, which is available to authorized users.

## Background

MicroRNAs (miRNAs) are some of non-coding RNAs with 20∼25 nucleotides [1]. In the process of post-transcription, miRNAs are a part of messenger RNA (mRNA) sequences and affect protein synthesis [2–4]. Some previous studies have proved that miRNAs are related to various diseases including cancers. For example, the expression level of *hsa*-*mir*-21 leads to more than 125 diseases, such as Alzheimer Disease, Diabetes Mellitus, Lymphoma and so on. Thus, the research of miRNAs is helpful for the diagnosis and treatment of diseases [5]. The traditional experiments to detect the associations between miRNAs and diseases are time-consuming and expensive [6]. Therefore, it is especially important to find potential miRNA-disease associations by the computational methods [7]. Previous researches achieved massive miRNA-disease associations through the traditional experiments, and some databases have been constructed for miRNA-disease associations. Human MicroRNA Disease Database (HMDD) [8] collects 572 miRNAs, 378 Disease and 10368 miRNA-disease associations. The miR2Disease [9] includes 349 miRNAs, 163 disease and 3273 miRNA-disease associations. The dbDEMC contains of 2224 miRNAs, 36 cancer types and 20037 miRNA-disease associations through the high-throughput methods. Thus, these associations promote the development of the computing methods.

Up to now, it has achieved excellent performance that people find the potential disease-miRNA associations by the computational methods [10–14]. Most of these methods are based on the assumption that miRNAs with high similarity apt to be related with similar diseases and vice versa [15, 16]. Xuan et al. [17] proposed HDMP that achieves a score for one miRNA by weighting *k* most similar neighbors, and a larger score has higher possibility to associate with a specific disease, but HDMP can’t work for a new disease without known related miRNAs. Jiang et al. [18] devised a hypergeometric distribution-based model to calculate the score of each miRNA for a specific disease, and the miRNA with larger score tend to cause this disease. Scores of above two methods are based on miRNA neighbor information, which ignores entire informations of miRNA similarity network. Many models find miRNA-disease associations based on the similarity networks [19–23]. Chen et al. developed the RWRMDA model [24], which uses the information of miRNA functional similarity network and known miRNA-disease association network, and utilizes the random walk model to find the potential miRNA-disease association. However, RWRMDA is faced with the same problem as HDMP, because of the initial nonzero vector. Therefore, Chen et al. [25] proposed WBSMDA to find the potential association by integrating the miRNA functional similarity network, disease semantic similarity and known miRNA-disease association network. For the similarity between two miRNAs/diseases, WBSMDA integrates Gaussian Interaction Profile (GIP) kernel similarity for miRNA and disease, and calculates the association probability for miRNA-disease pair using Within-Score and Between-Score of disease and miRNA. Gu et al. [26] developed NCPMDA by constructing novel similarity kernel for miRNA and disease via the matrix operation and calculating the space projection scores of miRNA and disease. The final score between miRNA and disease is calculated by combining two space projection scores. The predictive performance of NCPMDA is superior over the previous methods when working for a disease without any known related miRNAs [13].

Many previous models are based on defining a cost function and minimizing this cost function. Chen et al. [27] developed RLSMDA, a semi-supervised method, which minimizes the Regularized Least Squares cost function and uncovers the potential miRNAs associated with various diseases. After that, Chen et al. [28] proposed LRSSLMDA, which is used to reveal the potential association between miRNA and disease. LRSSLMDA constructs comprehensive statistical features and graph theoretic features by combining the miRNA and disease similarity kernels. Then, Laplacian regularization term is used to add objective function. Experimental results demonstrate that LRSSLMDA is a valuable computational model. In addition, many previous methods are based on machine learning algorithms [29, 30], matrix completion [31–33] and graph theory [34]. For example, Shen et al. [35] proposed CMFMDA that uses WKNKN to estimate association probability for unknown associations between miRNA and disease, and uses Collaborative Matrix Factorization to uncover the potential association. You et al. [36] developed PBMDA that constructs a heterogeneous graph by integrating five networks, gets all scores of paths for a miRNA-disease pair, and calculates the miRNA-disease association possibility through the sum of all path score. PBMDA gets a remarkable performance to find the potential miRNA-disease association.

All above methods have achieved remarkable results, but there are still different limitations or restrictions. For example, most of the existing methods are based on the assumption that miRNAs with high similarity apt to be related with similar diseases. About constructing miRNA and disease similarity kernel, most researches use the functional similarity and GIP kernel similarity for miRNA, and use the semantic similarity and GIP kernel similarity for disease. To integrate two similarity kernels, lots of works only tend to accumulate or average [29, 37, 38]. Therefore, there is an urgent need to propose an effective method for integrating multiple miRNA and disease similarity kernels [39].

In this paper, we firstly extract the miRNA functional similarity, the miRNA sequence similarity and GIP kernel similarity for miRNA, and the disease semantic similarity, disease functional similarity and GIP kernel similarity for disease. Then, we use the Fast Kernel Learning method to construct one miRNA similarity kernel and one disease similarity kernel. Finally, we propose a novel Sparse Laplacian Regularized Least Squares method to uncover the miRNA-disease association. Here, three evaluation methods are used to assess performance, including global Leave-One-Out Cross Validation (global LOOCV), local Leave-One-Out Cross Validation (local LOOCV) and 5-fold cross validation (5-fold CV). In these three evaluation methods, our method obtains the remarkable performance (AUCs of 0.9563, 0.8398 and 0.9535, respectively) compared with other nine models. And also, we use case studies of eight Neoplasms for further analyzing the performance of our method. We find that 47 of top 50 candidates are confirmed to have associations with Lymphoma in global verification, and all top 50 candidates are confirmed to have associations with Breast and Colorectal Neoplasms in local verification. Moreover, we find that some of the miRNAs need to be paid more attention to uncover more associations with various neoplasms, including hsa-mir-106b, hsa-mir-19b, hsa-mir-29c, hsa-mir-1, hsa-mir-29a and so on.

## Methods

**Fig. 1:**
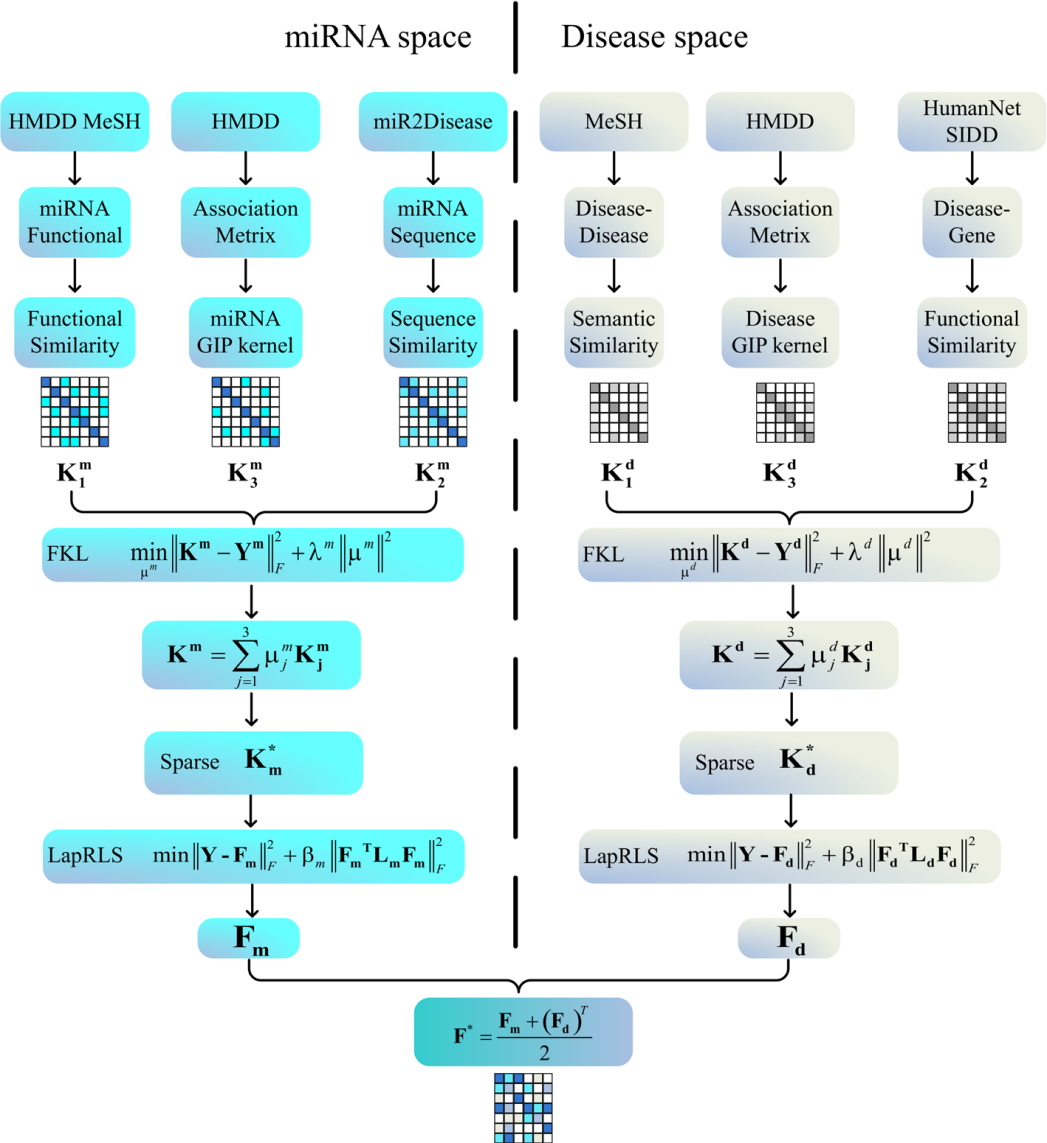
The flowchart of our method, FKL-Spa-LapRLS, for the miRNA-disease association prediction

We firstly use three miRNA similarity kernels and three disease similarity kernels to uncover potential miRNA-disease associations, respectively. Then, we combine these similarity kernels into a miRNA similarity kernel and a disease similarity kernel using Fast Kernel Learning, and sparse two similarity kernels after combination. Finally, we use Laplacian Regularized Least Squares to construct a loss function and get predicted association matrix from miRNA and disease space, respectively. Figure 1 is the flow chart of our method.

### Human miRNA-disease associations dataset

In this paper, the set of miRNAs is denoted by $M=\left \{m_{i}\right \}_{i=1}^{m}$, and the set of diseases is denoted by $D=\left \{d_{j}\right \}_{j=1}^{n}$, where *m* and *n* are the numbers of miRNAs and diseases respectively. The associations between miRNAs and diseases can be downloaded from HMDD database, which include 5430 associations between 495 miRNAs and 383 diseases. The associations are represented by a binary matrix *Y*∈*R*^*m*×*n*^, where *y*_*i*,*j*_∈{0,1}. if a miRNA *m*_*i*_ is association with a disease *d*_*j*_, *y*_*i*,*j*_ is set to 1; otherwise, *y*_*i*,*j*_ is set to 0;

### MiRNA similarity

Basing on the assumption that miRNAs with high similarity tend to be associated with the same disease, we extract three classes of miRNA similarity, including functional similarity, sequence similarity and Gaussian Interaction Profile (GIP) kernel similarity.

#### MiRNA functional similarity

In the previous works, the MISIM method [40] proposed by Cui et al. calculated the score of miRNA functional similarity. We extract 495 functional similarity score through MISIM and construct kernel $K_{1}^{m} \in R^{m\times m}$ to represent the miRNA functional similarity network, in which $K_{1}^{m}(m_{i},m_{j}$) is the functional similarity score between miRNAs *m*_*i*_ and *m*_*j*_.

#### MiRNA sequence similarity

All 495 miRNA sequences are downloaded from miRBase database [41]. We extract miRNA sequence similarity using the Needleman-Wunsch Algorithm and get kernel $K_{2}^{m}\in R^{m\times m}$ to represent the miRNA similarity of sequence network, in which $K_{2}^{m}(m_{i},m_{j})$ is the similarity of sequence score between miRNA *m*_*i*_ and *m*_*j*_.

#### GIP kernel similarity for miRNAs

GIP the kernel similarity [29, 38, 42] between miRNAs *m*_*i*_ and *m*_*j*_ is denoted as $K_{3}^{m}\in R^{m\times m}$ and the calculation method is as Eq. (1)

1 $$\begin{array}{@{}rcl@{}}  K_{3}^{m}(m_{i},m_{j})=exp\left(-\gamma_{m} \parallel IP(m_{i})-IP(m_{j})\parallel^{2}\right) \end{array} $$

where *IP*(*m*_*i*_)∈*R*^1×*n*^ denotes the interaction profiles of miRNA *m*_*i*_ by observing whether miRNA *m*_*i*_ is associated with each disease or not, that is to say, the *i*-th row of the associations matrix *Y*; *γ*_*m*_ is used for kernel bandwidth control, which is set to − 1 in this paper.

### Disease similarity

We extract three classes of disease similarity, including semantic similarity, functional similarity and GIP kernel similarity.

#### Disease semantic similarity

In the previous research [37, 40], disease *d*(*i*) can be described as a node in Directed Acyclic Graph(*DAG*) based on the MeSH [43] database (https://www.nlm.nih.gov/bsd/disted/meshtutorial/themeshdatabase/), and denoted as ${DAG}_{d_{i}}=(d_{i},T_{d_{i}},E_{d_{i}})$, in which $T_{d_{i}}$ is the set of all ancestor nodes of *d*_*i*_ including node *d*_*i*_ itself and $E_{d_{i}}$ is the set of corresponding links. A semantic score of each disease $t \in T_{d_{i}}$ can be calculated by Eq. (2).

2 $$  {}D_{d_{i}}(t) = \left\{ \begin{array}{lc} 1 & if ~t = d_{i} \\ max\left\{\Delta * D_{d_{i}}(t^{\prime}) |t^{\prime} \in children ~of ~t\right\} & if ~t \neq d_{i} \end{array} \right.  $$

where *Δ* is the semantic contribution factor, which is set to 0.5 in this paper.

Then, we define the semantic score of disease *d*_*i*_ by Eq. (3).

3 $$\begin{array}{@{}rcl@{}}  DV(d_{i}) = {\sum}_{t \in T_{d_{i}}} D_{d_{i}}(t) \end{array} $$

Therefore, we denote the disease semantic similarity as $K_{1}^{d}\in R^{n\times n}$ and the disease semantic similarity value between *d*_*i*_ and *d*_*j*_ is calculated by Eq. (4).

4 $$\begin{array}{@{}rcl@{}}  K_{1}^{d}\left(d_{i},d_{j}\right) = \frac{{\sum}_{t \in T_{d_{i}} \cap T_{d_{j}}} \left(D_{d_{i}}(t)+D_{d_{j}}(t)\right)}{DV(d_{i})+DV(d_{j})} \end{array} $$

#### Disease functional similarity

The associations between disease-gene and gene-gene are widely used to understand disease similarity [44]. From the HumanNet [45] database, we download the interactions of genes and one interaction has an log likehood score (LLS) that measure the probability of a functional linkage between genes. The LLS scores are normalized by Eq. (5)

5 $$\begin{array}{@{}rcl@{}}  LLS^{*}(g_{i},g_{j})=\frac{LLS(g_{i},g_{j})-{LLS}_{min}}{LLS_{max}-{LLS}_{min}} \end{array} $$

where *LLS*(*g*_*i*_,*g*_*j*_) represents LLS between the *i*-th and *j*-th genes; *LLS*^∗^(*g*_*i*_,*g*_*j*_) represents the LLS score after normalization; *LLS*_*min*_ and *LLS*_*max*_ indicate the minimum and maximum LLS scores in HumanNet respectively.

The functional similarity score between two genes is defined as Eq. (6)

6 $$  {}FS(g_{i},g_{j}) = \left\{ \begin{array}{lc} 1 & if ~i = j \\ LLS^{*}(g_{i},g_{j}) & if ~i \neq j ~and ~e(i,j) \in S_{HumanNET} \\ 0 & if ~i\neq j ~and ~e(i,j) \notin S_{HumanNET} \end{array} \right.  $$

where *S*_*HumanNET*_ indicates the gene-gene associations in the HumanNet database; *e*(*i*,*j*) indicates the association between *i*-th and *j*-th genes.

Then, the functional similarity score between a gene *g* and a gene set *G* is defined as Eq. (7).

7 $$\begin{array}{@{}rcl@{}}  F_{G}(g)= \max_{g_{i}\in G}(FS(g,g_{i})) \end{array} $$

In many cases, a disease *d*_*i*_ is related to many genes, which is defined as gene set *G*_*i*_, the associations between disease and genes are download from SIDD [46]. The disease functional similarity score is defined as Eq. (8)

8 $$\begin{array}{@{}rcl@{}}  K_{2}^{d}\left(d_{i},d_{j}\right)=\frac{\sum_{g_{k} \in G_{j}}F_{G_{i}}(g_{k})+\sum_{g_{s} \in G_{i}}F_{G_{j}}(g_{s})}{|G_{j}|+|G_{i}|} \end{array} $$

#### GIP kernel similarity for diseases

Similar to calculation of GIP kernel similarity for miRNA, GIP kernel similarity for disease is denoted as $K_{3}^{d}\in R^{n\times n}$, calculated as Eq. (9).

9 $$\begin{array}{@{}rcl@{}}  K_{3}^{d}\left(d_{i},d_{j}\right)=exp\left(-\gamma_{d} \parallel IP(d_{i})-IP(d_{j})\parallel^{2}\right) \end{array} $$

where *IP*(*d*_*i*_)∈*R*^*m*×1^ denotes the interaction profiles of disease *d*_*i*_ by observing whether disease *d*_*i*_ is associated with each miRNA or not, that is to say, the *i*-th column of the associations matrix *Y*; *γ*_*d*_ is used for kernel bandwidth control, which is set to − 1 in this paper.

### Fast kernel learning

Considering that a single similarity kernel cannot cover all information between miRNAs, we integrate $K_{1}^{m}$, $K_{2}^{m}$ for $K_{3}^{m}$ to get a new integrated similarity kernel *K*^*m*^∈*R*^*m*×*m*^ using the method of Fast Kernel Learning (FKL) [47]. We define *K*^*m*^ as Eq. (10).

10 $$\begin{array}{@{}rcl@{}}  K^{m}=\sum_{j=1}^{3} \mu_{j}^{m} K_{j}^{m} \end{array} $$

It is believed that *K*^*m*^ should be close to the associations metrix *Y*. We define the miRNAs associations similarity as Eq. (11).

11 $$\begin{array}{@{}rcl@{}}  Y^{m}=YY^{T} \end{array} $$

Therefore, we would like to find *μ*^*m*^∈*R*^3×1^ using the following Eq. (12) to minimize the distance between *K*^*m*^ and *Y*^*m*^.

12 $$\begin{array}{@{}rcl@{}}  \min \limits_{\mu^{m}} ||K^{m}-Y^{m}||_{F}^{2} \end{array} $$

where $||K^{m}-Y^{m}||_{F}^{2} = \sum _{i}\sum _{j}\left (K_{i,j}^{m}-Y_{i,j}^{m}\right)^{2}$.

To avoid overfitting in learning procedure, a regularization term should be added to equation as Eq. (13).

13 $$\begin{array}{@{}rcl@{}}  \begin{aligned} \min \limits_{\mu^{m}} &~~~||K^{m}-Y^{m}||_{F}^{2} + \lambda^{m}||\mu^{m}||^{2} \\ s.t. &~~~\mu_{j}^{m} \ge 0, j=1,2,3 \\ ~ &~~~\sum_{j=1}^{3} \mu_{j}^{m}=1 \end{aligned} \end{array} $$

where *λ*^*m*^ is set to 200 in this paper.

We use the matlab R2017a CVX to solve this optimization problem and obtain the integrate parameter $\mathcal {\mu }^{m} \in R^{1 \times 3} $ for miRNA functional similarity, miRNA sequence similarity and GIP kernel similarity. Therefore, the integrated miRNA similarity kernel is defined as Eq. (14).

14 $$\begin{array}{@{}rcl@{}}  K^{m}=\sum_{j=1}^{3} \mathcal{\mu}_{j}^{m} K_{j}^{m} \end{array} $$

Similarly, we obtain the integrate parameter $\mathcal {\mu }^{d} \in R^{1 \times 3} $ for disease semantic similarity, disease functional similarity and GIP kernel similarity by FKL, and the integrated disease similarity kernel is defined as Eq. (15).

15 $$\begin{array}{@{}rcl@{}}  K^{d}=\sum_{j=1}^{3} \mathcal{\mu}_{j}^{d} K_{j}^{d} \end{array} $$

### Laplacian regularized least squares

Given the similarity kernels of miRNAs and diseases, we use Sparse Laplacian Regularized Least Squares (Spa-LapRLS) to get a new association matrix, and find potential miRNA-disease associations. It includes Sparse kernel model and LapRLS model.

#### Sparse kernel model

We use a Top-*k* Neighbor model to reduce noise in integrated similarity kernel. For the miRNA subspace, we construct a weight matrix *w*_*m*_∈*R*^*m*×*m*^ for *K*^*m*^, whose elements are defined as Eq. (16), by the Top-*k* Neighbor method.

16 $$ w_{m}(i,j) = \left\{\!\! \begin{array}{lc} 1 & if~K^{m}(i,j) > \max(T(k,i),T(k,j)) \\ 0.5 &\! if~K^{m}(i,j)\! \in [\min(T(k,i),T(k,j)),\max(T(k,i),T(k,j))] \\ 0 & if~K^{m}(i,j) < \min(T(k,i),T(k,j)) \end{array}\right.  $$

where *k* satisfies condition 0<*k*<*m*; *T*(*k*,*i*) represents the *k*-th largest element of the *i*-th row in *K*^*m*^ and *T*(*k*,*j*) represents the *k*-th largest element of the *j*-th column in *K*^*m*^.

Therefore, we record the denoised miRNA similarity kernel as Eq. (17)

17 $$\begin{array}{@{}rcl@{}}  K_{m}^{*}=w_{m} \circ K^{m} \end{array} $$

Similarity, we also calculate the denoised disease similarity kernel as $K_{d}^{*} \in R^{n \times n}$.

#### LapRLS for miRNA-disease interaction prediction

Given a pair of similarity kernels for miRNA $K_{m}^{*}$ and disease $K_{d}^{*}$, we first use the Least Squares on the two subspace, and add Laplacian Regularization term to avoid overfitting. For miRNA subspace, the objective function of LapRLS [48] is defined as Eq. (18)

18 $$\begin{array}{@{}rcl@{}}  \min\limits_{F_{m}} ~||Y-F_{m}||_{F}^{2} + \beta_{m}||F_{m}^{T}L_{m}F_{m}||_{F}^{2} \end{array} $$

where $F_{m}=K_{m}^{*} \alpha _{m} \in R^{m \times n}$ is the predictive association matrix from miRNA; $L_{m} = D_{m}^{-\frac {1}{2}}\left (D_{m} -K_{m}^{*}\right)D_{m}^{-\frac {1}{2}} $, in which *D*_*m*_ is the diagonal matrix of $K_{m}^{*}$ in the form of $D_{m}(i,i)=\sum _{j=1}^{m}K_{m}^{*}(i,j)$; *β*_*m*_ is the regularization coefficients, which is set to 2^−5^ in this paper; *α*_*m*_ is renewed by the function Eq. (19) in [48].

19 $$  {}\alpha_{m}\,=\,\arg\!\! ~\min\limits_{\alpha_{m}\in R^{m \times n}} ~\!\! \left\{||Y\,-\,K_{m}^{*} \alpha_{m}\! ||_{F}^{2}\! +\! \beta_{m}||\alpha_{m}^{T}K_{m}^{*} L_{m}K_{m}^{*} \alpha_{m}||_{F}^{2}\right\}  $$

The derivation of the optimization algorithm are presented in [48].

In this way, the predicted associations matrix for all miRNA-disease pairs from the view of miRNAs are obtained as Eq. (20).

20 $$  F_{m} = K_{m}^{*}\left(K_{m}^{*}+\beta_{m}L_{m}K_{m}^{*}\right)^{-1}Y  $$

Similarly, we can get the predicted associations matrix for all miRNA-disease pairs from the view of miRNAs as Eq. (21)

21 $$  F_{d} = K_{d}^{*}\left(K_{d}^{*}+\beta_{d}L_{d}K_{d}^{*}\right)^{-1}Y^{T}  $$

where $F_{d}=K_{d}^{*} \alpha _{d} \in R^{n \times m}$; *β*_*d*_ is the regularization coefficients, which is set to 2^−5^ in this paper.

In the end, the predicted associations matrix from the view of miRNA and disease is defined as Eq. (22)

22 $$  F^{*} = \frac{F_{m}+F_{d}^{T}}{2}  $$

where *F*^∗^∈*R*^*m*×*n*^.

## Results and discussion

In this section, we study the performance of our method from different aspects on prediction of unknown miRNA-disease associations. First, we establish three evaluation methods and two assessment indicators to evaluate the accuracy of our method. Second, we analyze the performance of our method with different parameters by using 10-fold CV and local LOOCV. Third, we employ 10-fold CV and local LOOCV to analyze the performance of the FKL model. Fourth, we compare the performance of LapRLS with multiple matrix factorization method. Fifth, we compare the performance of FKL-Spa-LapRLS with nine outstanding methods. Finally, for a further validation, we implement the global and local verifications on eight neoplasms for case studies.

### Evaluation criteria

In this paper, we implement 10-fold CV, global LOOCV and local LOOCV to evaluate the prediction accuracy of our method. In the 10-fold CV, all miRNA-disease associations are randomly divided into ten uncrossed groups, one of which is regarded as test set and the other nine groups are used for training set in turns. In the global LOOCV, all 5430 miRNA-disease verified associations are regarded as objective research sample, and each association is left in turns served as a test sample and other known associations are regarded as training sample. In the local LOOCV, only considering miRNAs for a specific disease, for disease *d*(*i*), each miRNA related to *d*(*i*) is left out as test set, and other associations are regarded as training set. All the miRNA-disease associations in test set are reseted as 0 in the association matrix *Y*.

In our study, we use Area Under Curve (AUC) and Area Under the Precision-Recall curve (AUPR) to establish the assessment criteria for method prediction. AUC is the area under the receiver operating characteristic (ROC) created by plotting true positive rate against false positive rate at various threshold settings. An AUC value of 1 indicates perfect performance and an AUC of 0.5 indicates random performance. AUPR is the area under the curve created by plotting precision against recall at various threshold setting. The greater the value of AUPR, the better performance of the model.

### Parameter selection

In this section, we use 10-fold CV and local LOOCV to analyze several parameters, including *γ*_*m*_, *γ*_*d*_, *λ*_*m*_, *λ*_*d*_, *β*_*m*_, *β*_*d*_ and *k* value.

**Fig. 2:**
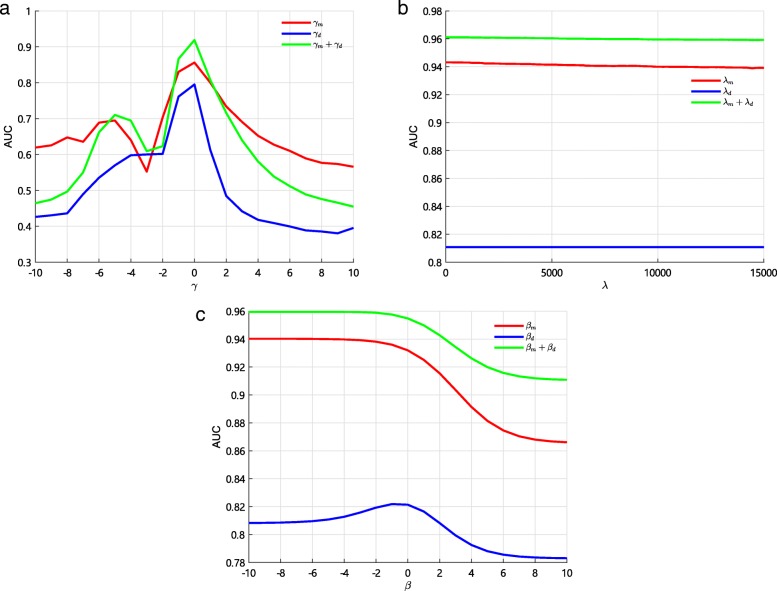
The AUCs of parameters by the 10-fold CV. Blue line and red line represent the AUCs of using single kernel. Green line represents the AUCs of using two kernels. **a** The AUCs of GIP with different *γ*. **b** The AUCs of FKL with different *λ*. **c** The AUCs of LapRLS with different *β*

The *γ*_*m*_ and *γ*_*d*_ are the parameters in the process of constructing GIP kernel similarity for miRNA and diseases, respectively. We just use GIP kernel similarity to predict potential miRNA-disease associations and use 10-fold CV to evaluate performance of GIP kernel with different parameters. Then, we take *γ*_*m*_ and *γ*_*d*_ from − 10 to 10 with step 1 and calculate AUCs, respectively. The results are shown in Fig. 2a. It shows that the performance of GIP similarity kernel is sensitive to *γ*_*m*_ and *γ*_*d*_, and the optimal AUC is obtained when *γ*_*m*_ and *γ*_*d*_ equal to 0. However, the *K*_*m*,3_ and *K*_*d*,3_ are matrices with ones in all elements according to Eqs. (1) and (9) when two parameters equal to 0. Therefore, we adopt suboptimal *γ*_*m*_=−1 and *γ*_*d*_=−1 in this paper. Since most of elements in GIP similarity kernel are more than 1, we need to normalize GIP similarity kernel before integrating multiple kernels.

The *λ*_*m*_ and *λ*_*d*_ are the regularization coefficients of FKL. We use different *λ*_*m*_ and *λ*_*d*_ to integrate three miRNA similarity kernels and three disease similarity kernels, respectively. Then we use integrated similarity kernel and LapRLS to uncover potential associations and use 10-fold CV to evaluate performance of FKL with different parameters. The *λ*_*m*_ and *λ*_*d*_ are gradually varying from 0 to 15000 with step 100 in order to find the best value. The results are shown in Fig. 2b. It can be found that AUC keeps small fluctuation in the range between 0 to 15000. It demonstrates that FKL is insensitive to regularization coefficient. So, *λ*_*m*_ and *λ*_*d*_ are set to 200 in this paper.

The *β*_*m*_ and *β*_*d*_ are the regularization coefficients of LapRLS. We take *β*_*m*_ and *β*_*d*_ from 2^−10^ to 2^10^, respectively. We adopt 10-fold CV to evaluate performance of LapRLS with different parameters. The results are shown in Fig. 2c. It can be found that AUC keeps small fluctuation in the range between 2^−10^ to 2^−2^, and AUC has obvious change when *β*_*m*_ and *β*_*d*_ greater than 2^−2^. We select the optimal *β*_*m*_ and *β*_*d*_ by the highest AUC value and set *β*_*m*_ and *β*_*d*_ as 2^−5^ in this paper.

**Fig. 3:**
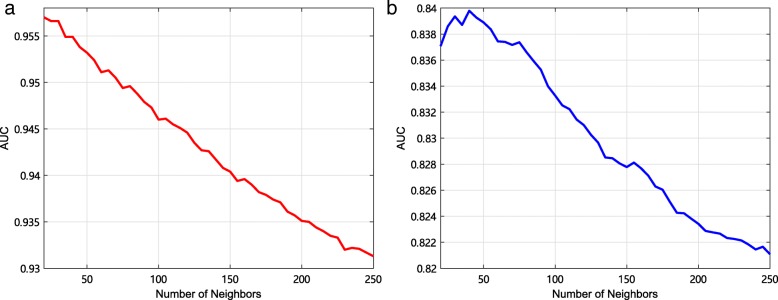
The results of our method with different *k* values. **a** The AUCs of LapRLS with different *k* by the 10-fold CV. **b** The AUCs of LapRLS with different *k* by the local LOOCV

Meanwhile, *k* value in the process of sparse kernel is an important parameter in this paper. We use 10-fold CV and local LOOCV to analyze *k* value. The value of *k* is taken from 20 to 250 with step 5, are shown in Fig. 3. It can be clearly seen that the process of sparse kernel has positive effect on the discovery of potential miRNA-disease associations. In this study, *k* value is set to 20 in the 10-fold CV and global LOOCV, and is set to 40 in the local LOOCV.

### FKL performance analysis

In this section, we analyze the performance of FKL. First, we compare FKL with single kernel and average kernel by the 10-fold CV and local LOOCV. Then, we compare FKL with two multiple kernels learning method by the 10-fold CV and local LOOCV.

#### Comparison with single kernel and average kernel

We compare the prediction performance of FKL with three single similarity kernels and an average similarity kernels by using 10-fold CV and local LOOCV methods. The experiments are remarked as following.

23 $$ {} \left\{ \begin{array}{lc} K_{1}^{m} ~~\& ~~K_{1}^{d} &K_{1}\\ K_{2}^{m} ~~\& ~~K_{2}^{d} &K_{2}\\ K_{3}^{m} ~~\& ~~K_{3}^{d} &K_{3}\\ avg\left(K_{1}^{m},K_{2}^{m},K_{3}^{m}\right) ~~\& ~~avg\left(K_{1}^{d},K_{2}^{d},K_{3}^{d}\right) &AVG\\ K_{m}^{*} ~~\& ~~K_{d}^{*} & FKL \end{array} \right.  $$

**Fig. 4:**
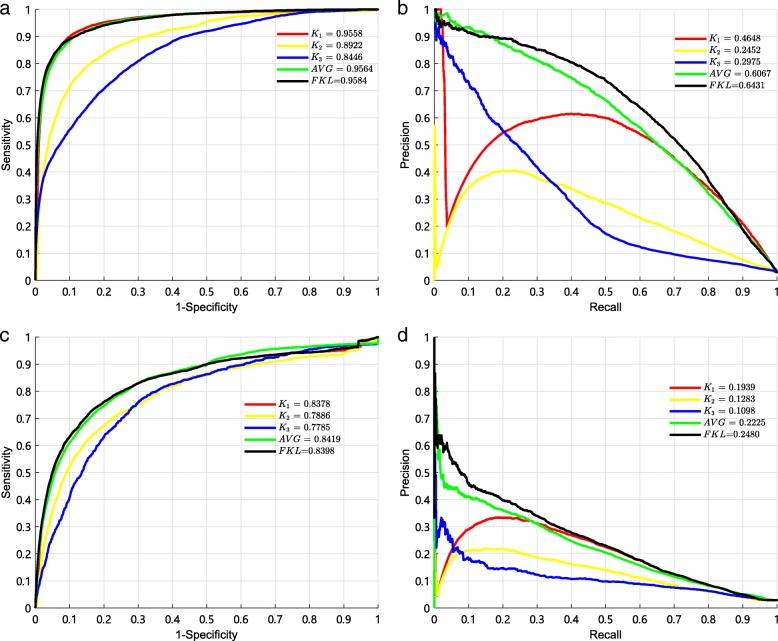
The AUCs and AUPRs of five models by the 10-fold CV and local LOOCV. **a** The AUCs of five models by the 10-fold CV. **b** The AUPRs of five models by the 10-fold CV. **c**: The AUCs of five models by the local LOOCV. **d** The AUPRs of five models by the local LOOCV

The comparison results obtained by the 10-fold CV and local LOOCV are shown in Fig. 4.

In the 10-fold CV, The AUC of FKL is the highest among five curves, and the AUC difference between the FKL model and the *K*_1_ is slight but the difference in AUPR is obvious. Local LOOCV is a measure that can express model performance excellently when we handle a new disease not having known associations with miRNA. In Fig. 4, the *AUC* of average kernel is greater than FKL kernel. In the process of KFL, we need to find a optimized *μ* to weight kernels. Here, we get $\mathcal {\mu }^{m}=\left (0.6610,0.3390,1.1562\times 10^{-9}\right)$ and $\mathcal {\mu }^{d}=\left (1,9.1453\times 10^{-10},7.3854\times 10^{-10}\right)$, that is to say, the miRNA functional similarity kernel and the miRNA sequence similarity kernel are more important than GIP kernel similarity, and disease semantic similarity kernel is the most important in the three kernels. The model loses a part of information in the weighting process. However, a new disease not having any known association with miRNA needs more detail information from different aspects. The average kernel method satisfies this requirement of more detail informations. That is why the AUC of FKL model is lower than average kernel, but the AUPR of FKL model is higher than average kernel method. Moreover, AUPR can evaluate the classifier performance better when dealing with unbalanced dataset. Therefore, it demonstrates that the FKL model is most significant in all kinds of models.

#### Comparison with other multiple kernel learning methods

**Fig. 5:**
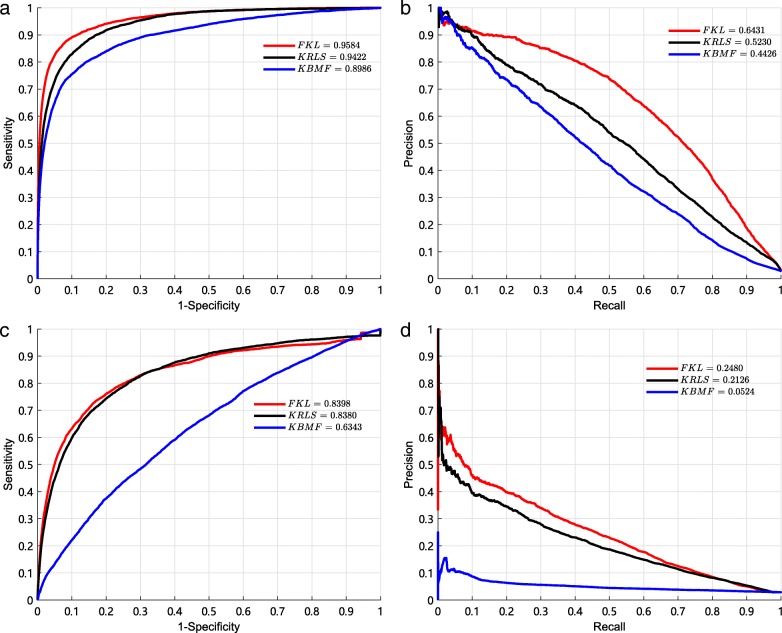
The AUCs and AUPRs of three multiple kernel learning methods by the 10-fold CV. **a** The AUCs of three models by the 10-fold CV. **b** The AUPRs of three models by the 10-fold CV. **c** The AUCs of three models by the local LOOCV. **d** The AUPRs of three models by the local LOOCV

Several multiple kernel learning methods have been proposed to predict microRNA-disease associations, including Kronecker regularized least squares (KRLS) [39, 49] and kernelized Bayesian matrix factorization (KBMF) [32, 50]. We compare FKL with these two methods to integrate the similarity kernels to predict potential associations, respectively. Then, we use 10-fold CV and local LOOCV to evaluate performance of these three methods. The comparison results are shown in Fig. 5. In the 10-fold CV, it can be observed that the best AUC of 0.9584 and the best AUPR of 0.6431 are obtained by FKL. Comparing with KRLS, FKL achieves AUC improvement of 0.0162 (0.9584 over 0.9422) and AUPR improvement of 0.1201 (0.6431 over 0.5230). Comparing with KBMF, FKL achieves AUC improvement of 0.0598 (0.9584 over 0.8986) and AUPR improvement of 0.2005 (0.6431 over 0.4426). In local LOOCV, it can be observed that the best AUC of 0.8398 and the best AUPR of 0.2480 are also obtained by FKL. It shows that FKL is excellent at the aspect of uncovering associations between miRNAs and diseases.

### Comparison with matrix factorization

**Fig. 6:**
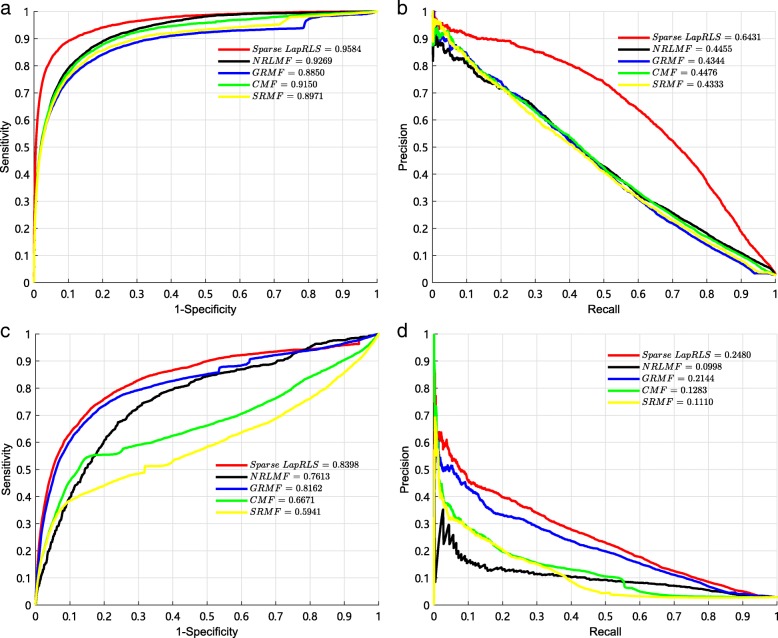
The comparison results between our method and other matrix factorization models by the 10-fold CV and local LOOCV. **a** The AUCs of five models by the 10-fold CV. **b** The AUPRs of five models by the 10-fold CV. **c** The AUCs of five models by the local LOOCV. **d** The AUPRs of five models by the local LOOCV

The matrix factorization (MF) methods are widely used for different bioinformatics applications, including Protein-Protein interactions (PPI) prediction, drug-target interaction (DTI) prediction, drug response prediction, and so on. Therefore, we compare sparse LapRLS with four MF methods, including Similarity-Regularized Matrix Factorization(SRMF) [51], Collaborative Matrix Factorization (CMF) [52], Neighborhood Regularized Logistic Matrix Factorization (NRLMF) [53] and Graph Regularized Matrix Factorization (GRMF) [54]. We use the same integrated similarity kernels and these five methods to predict potential associations, and adopt 10-fold CV to evaluate performance of different methods. The results are shown in Fig. 6. In 10-fold CV, it can be observed that the best AUC of 0.9584 and the best AUPR of 0.6431 are obtained by spa-LapRLS. In local LOOCV, it can be observed that the best AUC of 0.8398 and the best AUPR of 0.2480 are also obtained by sparse LapRLS. It demonstrates that sparse LapRLS is reliable for predicting miRNA-disease associations.

### Comparison with other methods

We furtherly compare the performance of FKL-Spa-LapRLS with nine computational prediction models (i.e., PBMDA [36], MCMDA [31], MaxFlow, NCPMDA [26], WBSMDA [25], HDMP [17], RLSMDA [27], LRSSLMDA [28], HGIMDA [55]), and the comparisons are shown in Table 1. In the local LOOCV, FKL-Spa-LapRLS gets an AUC of 0.8398, which is slightly under performance of NCPMDA (0.8584) and LRSSLMDA (0.8418). However, in the global LOOCV, our method gets an AUC of 0.9563, which is significant superior to the result of other methods. In the 5-fold, FKL-Spa-LapRLS obtains an AUC of 0.9535, which also has a great outperformance than other methods. Therefore, FKL-Spa-LapRLS improves the prediction performance of disease-miRNA associations from different evaluation measures.

**Table 1:** The comparison results between our method and other nine computational models

Methods	Global LOOCV	Local LOOCV	5-fold CV
**FKL-Spa-LapRLS**	**0.9563**	0.8398	**0.9535**
PBMDA	0.9169	0.8341	0.9172
MCMDA	0.8749	0.7718	0.8767
MaxFlow	0.8624	0.7774	0.8579
NCPMDA	0.9073	**0.8584**	0.8763
WBSMDA	0.8030	0.8031	0.8185
HDMP	0.8366	0.7702	0.8342
RLSMDA	0.8426	0.6953	0.8569
LRSSLMDA	0.9178	0.8418	0.9181
HGIMDA	0.8781	0.8077	—

The boldface is the best value in the column

### Case studies

In this section, we study several important diseases to further validate the predictive power of our method. We utilize the known miRNA-disease associations included in HMDD to find the potential miRNA-disease associations not included in HMDD, and verify the predicted results though two independent databases (dbDEMC [56] and miR2Disease [9]). In fact, dbDEMC and miR2Disease are commonly utilized to be benchmark datasets for many models, such as PBMDA and LRSSLMDA. The dbDEMC database includes 2224 miRNAs, 36 cancer types and 20037 miRNA-disease associations by the high-throughput method, and our model predicts the top five disease, including Colon Neoplasms, Gastric Neoplasms, Pancreatic Neoplasms, Colorectal Neoplasms and Esophageal Neoplasms. Furthermore, in previous work, Kidney Neoplasms, Breast Neoplasms and Lymphoma were used to infer their underlying associated miRNAs. Therefore, we use case studies of eight diseases to analyze the performance of FKL-Spa-LapRLS in this section.

We implement two methods, global validation and local validation, to evaluate the predicted performance of our method in case studies. In global verification, 5430 known miRNA-disease associations in HMDD are used as a training set to discover the potential associations. For each disease, we extract top 50 candidate associations that can’t be covered by training set. And we get all of 400 candidate associations that are checked by dbDEMC and miR2Disease databases. In the local validation, all known associations that are related to a special disease are reset to unknown ones. We use other known associations as training set to discover the potential associations. we also extract top 50 candidate associations for this special disease. And we obtain all of 400 candidate associations that are checked by the HMDD, miR2Disease and dbDEMC databases.

The verification results of eight diseases are listed in Table 2. In Table 2, the global verification is the number of confirmed associations by dbDEMC and miR2Disease in top 50 miRNAs. And the local verification is the number of identified associations by HMDD, dbDEMC and miR2Disease. In Table 2, we can find that 47 of top 50 candidates are associated with lymphoma confirmed by global verification, and local verification confirms that all top 50 candidates are associated with breast and Colorectal Neoplasms.

**Table 2:** The verification results about eight neoplasms types

Disease name	Global verification	Local verification
Colon Neoplasms	44	48
Gastric Neoplasms	42	40
Pancreatic Neoplasms	45	50
Colorectal Neoplasms	45	50
Esophageal Neoplasms	39	46
Kidney Neoplasms	43	43
Breast Neoplasms	39	50
Lymphoma	47	48

**Fig. 7:**
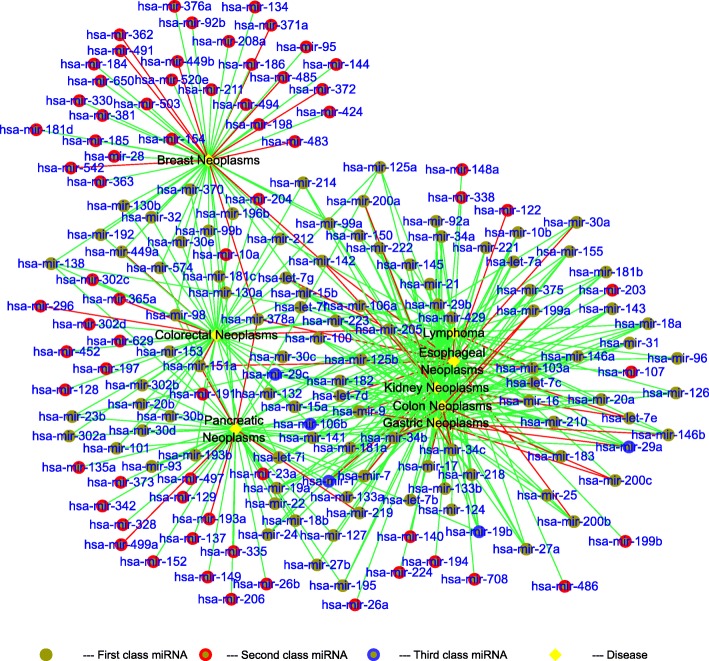
The case studies by global verification. The green lines are the confirmed candidate associations. The red lines are the unconfirmed candidate associations. The black nodes are the disease. The brown nodes are the candidate miRNAs. First class miRNA represents miRNA associated with multiple diseases. Second class miRNA represents miRNA associated with one disease. Third class miRNA represents important miRNA associated with more than six diseases

**Fig. 8:**
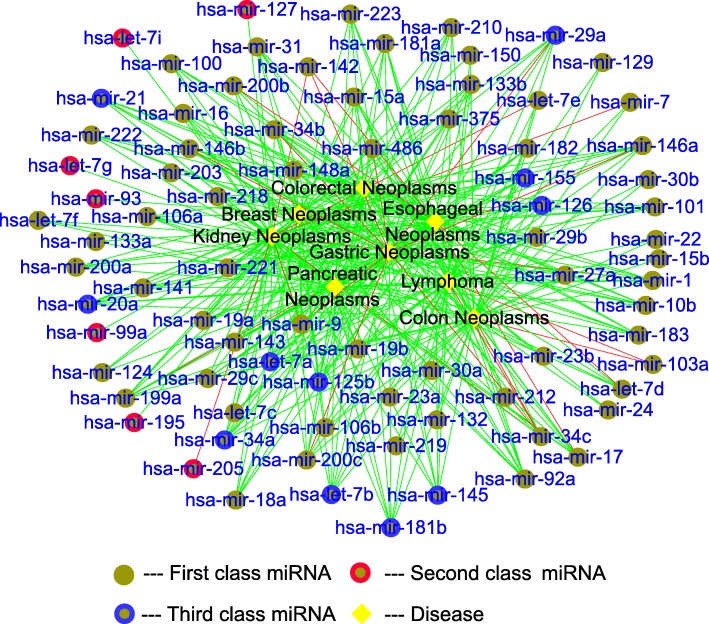
The case studies by local verification. The green lines are the confirmed candidate associations. The red lines are the unconfirmed candidate associations. The black nodes are the disease. The brown nodes are the candidate miRNAs. First class miRNA represents miRNA associated with multiple diseases. Second class miRNA represents miRNA associated with one disease. Third class miRNA represents important miRNA associated with more than six diseases

The results of case studies and some special miRNAs are shown in Figs. 7 and 8 (detail results in Additional files 1, 2, 3, 4, 5, 6, 7 and 8). The green lines are the confirmed miRNA-disease associations, the red lines are the unconfirmed miRNA-disease associations, the black nodes are the eight neoplasms, and the brown nodes are the predicted miRNAs associated with diseases. There are 400 associations in Fig. 7, and we can find that most of the miRNA-disease associations are confirmed by the global verification. In addition, there are many miRNAs that are only related to Breast Neoplasms but they have nothing to do with other diseases. And there are nine associations are unconfirmed. The reason is that of total 495 miRNAs in the training set, 202 have been linked to Breast Neoplasms, so there is a large possibility that the remaining miRNAs have no association with it. Similarly, there are 11 miRNAs related to Esophageal Neoplasms but not confirmed. The reason is that there are already 74 miRNAs associated with the Esophageal Neoplasms in the training set. On the other hand, there are a few unconfirmed miRNAs associated with other six diseases. In Fig. 7, we can see that hsa-mir-106b, hsa-mir-19b and hsa-mir-29c are associated with six out of eight diseases, and these miRNAs should be paid more attention to reveal more associations. Moreover, hsa-mir-1 and hsa-mir-29a are expected to be associated with five diseases out of eight diseases, but these associations still have not been verified by valid experiment. In Fig. 8, we can find that most of miRNAs work on various diseases. For a special disease with unknown associations with miRNAs, our method can reveal the miRNAs associated with it, and only 26 associations out of 400 cannot be confirmed by known experiments. These unconfirmed associations need to be paid more attention. Especially for hsa-let-7a, hsa-let-7b, hsa-mir-125b, hsa-mir-126, hsa-mir-145, hsa-mir-155, hsa-mir-181b, hsa-mir-20a, hsa-mir-21, hsa-mir-34a, hsa-mir-92a, these miRNAs are associated with all diseases. And we find that the related miRNAs among eight Neoplasms are highly similar. Therefore, it is very important to find more diseases related to these n11 miRNAs.

## Conclusions

In this paper, we propose a FKL-Spa-LapRLS model to uncover potential miRNA-disease associations. We demonstrate that the KFL model is more importance than the average kernel method using 10-fold CV and local LOOCV, and the process of sparse kernal has a positive effect on noise elimination in similarity network. The LapRLS method contributes to accuracy of finding potential miRNA-disease associations.

FKL-Spa-LapRLS has been compared with nine prediction methods that have got excellent performance for prediction of miRNA-disease associations, including PBMDA, MCMDA, MaxFlow, NCPMDA, WBSMDA, HDMP, RLSMDA, LRSSLMDA and HGIMDA. FKL-Spa-LapRLS has the significantly highest accuracy in 5-fold CV and global LOOCV, albeit weakly lower than NCPMDA and LRSSLMDA in local LOOCV. To further analyze the performance of FKL-Spa-LapRLS, we implement case studies of eight Neoplasms. We find that 47 of top 50 candidates are confirmed to be associated with Lymphoma in global verification and all the top 50 candidates are confirmed to be associated with Breast and Colorectal Neoplasms in local verification, and some miRNAs need to be paid more attention.

Of course, FKL-Spa-LapRLS also have some limitations that need to be improved in the future. For example, our method needs more similarity kernels that are constructed by many information about gene-disease, disease-disease and miRNA-miRNA, and it would lose some detail information in the process of FKL when handling a new disease without the known associations with miRNAs.

## Additional files

Additional file 1 Table S1. The top 50 predicted miRNAs related to Colon Neoplasms. (XLSX 11 kb)

Additional file 2 Table S2. The top 50 predicted miRNAs related to Gastric Neoplasms. (XLSX 10 kb)

Additional file 3 Table S3. The top 50 predicted miRNAs related to Pancreatic Neoplasms. (XLSX 11 kb)

Additional file 4 Table S4. The top 50 predicted miRNAs related to Colorectal Neoplasms. (XLSX 10 kb)

Additional file 5 Table S5. The top 50 predicted miRNAs related to Esophageal Neoplasms. (XLSX 11 kb)

Additional file 6 Table S6. The top 50 predicted miRNAs related to Kidney Neoplasms. (XLSX 10 kb)

Additional file 7 Table S7. The top 50 predicted miRNAs related to Breast Neoplasms. (XLSX 11 kb)

Additional file 8 Table S8. The top 50 predicted miRNAs related to Lymphoma. (XLSX 11 kb)

## Abbreviations

CMF: Collaborative matrix factorization
CV: Cross validation
FKL: Fast kernel learning
GIP: Gaussian interaction profile
GRMF: Graph regularized matrix factorization
HMDD: Human microRNA disease database
KBMF: Kernelized Bayesian matrix factorization
KRLS: Kronecker regularized least squares
LapRLS: Laplacian regularized least squares
LLS: Log likehood score
LOOCV: Leave-one-out cross validation
NRLMF: Neighborhood regularized logistic matrix factorization
SRMF: Similarity-regularized matrix factorization
